# Bi-alignments with affine gaps costs

**DOI:** 10.1186/s13015-022-00219-7

**Published:** 2022-05-16

**Authors:** Peter F. Stadler, Sebastian Will

**Affiliations:** 1 Bioinformatics Group, Department of Computer Science, and Interdisciplinary Center for Bioinformatics, Universität Leipzig, Härtelstraße 16–18, 04107 Leipzig, Germany; 2 Competence Center for Scalable Data Services and Solutions Dresden/Leipzig, Interdisciplinary Center for Bioinformatics, German Centre for Integrative Biodiversity Research (iDiv), and Leipzig Research Center for Civilization Diseases, Universität Leipzig, Augustusplatz 12, 04107 Leipzig, Germany; 3 Max Planck Institute for Mathematics in the Sciences, Inselstraße 22, 04109 Leipzig, Germany; 4 Department of Theoretical Chemistry, University of Vienna, Währinger Straße 17, 1090 Vienna, Austria; 5 Facultad de Ciencias, Universidad National de Colombia, Sede Bogotá, Ciudad Universitaria, 111321 Bogotá, D.C., Colombia; 6 Santa Fe Institute, 1399 Hyde Park Rd., Santa Fe, NM 87501 USA; 7 AMIBio, Laboratoire d’Informatique de l’École Polytechnique (LIX), Institute Polytechnique de Paris (IP Paris), Batiment Turing, 1 rue d’Estienne d’Orves, 91120 Palaiseau, France

**Keywords:** Dynamic programming, Scoring functions, Multi-tape formal grammar, Recursion

## Abstract

**Background:**

Commonly, sequence and structure elements are assumed to evolve congruently, such that homologous sequence positions correspond to homologous structural features. Assuming *congruent* evolution, alignments based on sequence *and* structure similarity can therefore optimize both similarities at the same time in a single alignment. To model *incongruent* evolution, where sequence and structural features diverge positionally, we recently introduced *bi-alignments*. This generalization of sequence and structure-based alignments is best understood as alignments of two distinct pairwise alignments of the same entities: one modeling sequence similarity, the other structural similarity.

**Results:**

Optimal bi-alignments with affine gap costs (or affine shift cost) for two constituent alignments can be computed exactly in quartic space and time. Even bi-alignments with affine shift and gap cost, as well as bi-alignment with sub-additive gap cost are optimized efficiently. Affine gap-cost bi-alignment of large proteins ($\sim 930$ aa) can be computed.

**Conclusion:**

Affine cost bi-alignments are of practical interest to study shifts of protein sequences and protein structures relative to each other.

**Availability:**

The affine cost bi-alignment algorithm has been implemented in Python 3 and Cython. It is available as free software from https://github.com/s-will/BiAlign/releases/tag/v0.3 and as bioconda package bialign.

**Supplementary Information:**

The online version contains supplementary material available at 10.1186/s13015-022-00219-7.

## Introduction

### Incongruent evolution

While biological function is eventually encoded in a genomic sequence, it relies on the “decoding” of the sequence into a spatially structured RNA or protein, or into specific interactions, such as the binding of a DNA element by a transcription factor. Natural selection acts to conserve function over evolutionary times and therefore preserves functional RNA or protein structures, binding motifs, intron–exon boundaries, etc. Stabilizing selection on such a functional entity typically also causes the conservation of its encoding DNA sequence. Homologous functional units, i.e. those that share a common ancestry [1], are therefore represented by homologous sequences. As a consequence, functional elements often can be identified based on their similarity in sequence alignments. For RNA and proteins, this allows the detection of consensus structures [2, 3], enables the identification of transcription factor binding sites [4], and the detection of conserved (non-coding) transcripts through the conservation of splice junctions [5].

Homology of a feature or trait, however, does not require that all its constituent parts are homologous. Most obviously, insertions and deletions in a DNA sequence imply that not all nucleotides trace back to a common ancestor even if the sequence as a whole does. Similarly, homology of a structural feature does not imply that all its constituent contacts are preserved. There are indeed well-documented exceptions to the by far most common case of homologous features being produced from homologous sequence positions. A well-studied, albeit apparently rare, example is *intron-sliding*, where the start and end of an intron “moves” in the same direction for the same number of nucleotides [6–9]. While the gene product is perfectly preserved, except possibly for some changes of the amino acids encoded by the few nucleotides involved in the sliding, both splice junctions are now encoded by non-homologous genomic positions. Promotors sometime exhibit a similar form of turnover, where a short binding site pattern at one site is replaced by the emergence of a matching sequence nearby [10]. In the context of biopolymer structures it is possible that contacts between nucleotides or amino acids are shifted relative to the underlying sequence in a way that preserves most features of the ancestral structure. Such transitions can be facilitated by the existence of kinetically accessible structural alternatives [11], of which different variants are stabilized by subsequent mutations in different lineages. In a preliminary survey, we recently observed that 72 of 1181 moderate-size Rfam families show evidence for this kind of incongruence between sequence and structure conservation [12]. This observation suggests that incongruent evolution of sequence and structure is relatively rare but still occurs with sufficient frequency to be non-negligible.

**Fig. 1:**
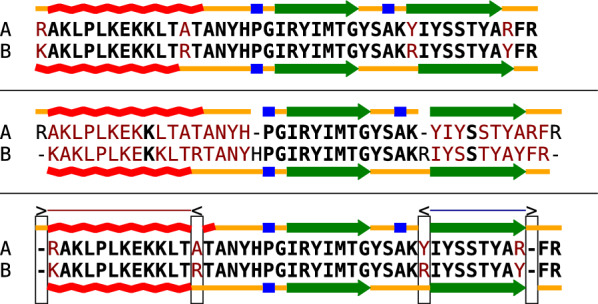
Two pairwise alignments and a bi-alignment of peptide sequences and their predicted secondary structures (helix red, turn blue, $\beta$-sheet green, coil orange). Structure are predicted according to the Chou Fasman method [13] with CFSSP [14]. To facilitate quick visual assessment of sequence alignment quality, sequence mismatches are shown in bold black, sequence indels in non-bold black, and mismatches in dark red. The upper alignment optimizes sequence similarity, and shows the structure out of sync: the helix is moved to left, the last $\beta$-sheet is shifted to the right by 1 position. The second alignment maximizes structural similarity and thus shows little sequence similarity. The evolution of the two peptides is explained much better by a bi-alignment (third panel), which supports shift events (marked by rectangles) that can shift either sequence against its structure to the left ($\texttt {<}$) or to the right ($\texttt {>}$). The resulting *regions of shift* are indicated by in general *k* blue and red lines corresponding to shifts by *k* positions to the left or to the right. While the shift events shown in this example delete and insert structure of A with respect to both sequences and the structure of B, shift alignments also support as well analogous shifts of sequences and the second structure (which would be shown in the bottom row). In our representation, shift events are the only visible difference between the bi-alignment $\mathbb {A}$ in the third panel and the two alignments. Nevertheless, the representation can be mapped to our formalization of bi-alignments as alignments of two constituent alignments $\mathbb {U}$ and $\mathbb {V}$: $\mathbb {U}$ is obtained from the 2nd and 3rd bi-alignment row by removing the two all-gap columns (i.e. the first and the 3rd-to-last column). The secondary structure alignment $\mathbb {V}$ coincides with the 1st and 4th row since there is no column that contains only gaps in these to rows

**Fig. 2:**
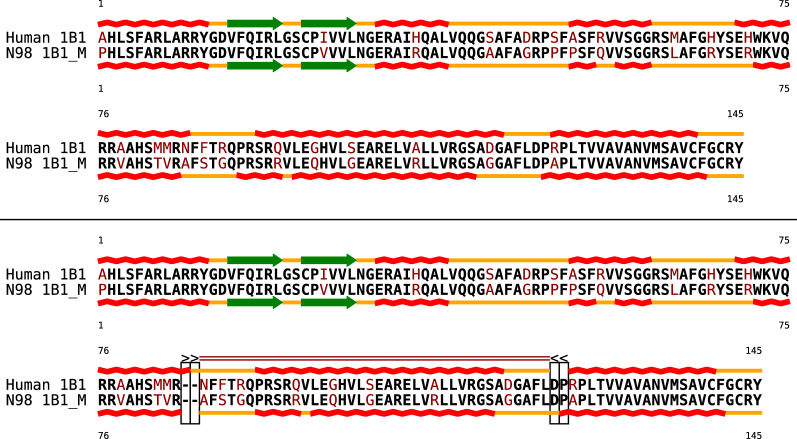
Alignment (top) and Bi-alignment (bottom) of 145 N-terminal amino acids of two CYP1B1 cytochrome P450 enzymes: the extant human enzyme (Human 1B1) and the corresponding ancestral mammalian cytochrome (N98 1B1_M). See Fig. 1 for the representation of the alignments and secondary structure elements. Only the bi-alignment properly aligns the ’shifted’ fifth helix and explains the structural incongruence by evolutionary shifts (two forward and two backward shifts

To our knowledge, incongruences between conserved protein sequence and conserved protein structures so far have not been studied systematically. However, the example of Fig. 1 demonstrates that (at least at the level of secondary structures) it is not at all difficult to obtain incongruence by performing a few mutations. Here, we (artificially) introduced substitutions into a peptide sequence such that predicted secondary structures shifted relative to the reference sequence. The comparative analysis of proteins occasionally reveals examples of natural incongruences between sequence and secondary structure; moreover, it shows that the phenomenon occurred at least occasionally in protein evolution. Figure 2(top) depicts the alignment of the extant human CYPB1 cytochrome P450 enzyme and its reconstructed ancestral mammalian counterpart, which was recently crystallized (PDB: 6OYU and 6OYV) and characterized functionally [15]. Despite the high level of similarity of the ancestral and extant folds, the bi-alignment (Fig. 2, bottom) reveals some differences in the extent of helices and suggests a shift of “helix D” by two amino acids, constituting an incongruence of the considered type. Another published example can be found in Fig. 5 of [16]: relative to the underlying sequence, one observes several small helix shifts in the evolution of the Pgp protein (MDR1) between human, mouse, and rat.

Incongruences between sequence homology and homology of structure or functional elements are rooted in the inherent redundancy of genotype-phenotype maps. For both RNA and proteins, very different sequences can encode the same fold or function [17–19], while at the same time identical sequences can appear in very different structural or functional contexts [20–22]. Together, these features sometimes lead to a sliding or migration of a functionally relevant structure in response to a fortuitously placed mutation. The occasional emergence of incongruences between sequence conservation and the conservation of structure thus is an expected consequence of the redundancies inherent in the sequence/structure relationship of biopolymers. It becomes a relevant empirical question, therefore, how frequent this process has been throughout evolutionary history.

Not only is incongruent evolution of interest as an under-studied aspect of evolutionary dynamics, but it has practical implications for data analysis. Incongruences impact our ability to detect and reconstruct consensus structures, since corresponding structural features are formed by evolutionarily unrelated nucleotides or amino acids, while homologous sequence positions form disparate structural elements. This means that (in the presence of incongruent evolution) a single multiple sequence alignment cannot simultaneously represent the similarities of sequence and structure. In particular, conserved structure can no longer be represented as ‘consensus structure’, i.e. as an annotation of the columns of a sequence alignment.

### Bi-alignments

We recently introduced bi-alignments [12, 23] as a mathematically consistent way of describing incongruent evolutionary relationships. Bi-alignments are motivated by treating shifts between sequence and structure explicitly as evolutionary events. It is important to realize that it is not necessarily possible to find an optimal reconciliation of sequence and structure alignments by identifying shifts events *a posteriori* from a pair of sequence and structure alignments that have been computed *separately*. Instead, bi-alignments allow *simultaneously* predicting sequence and structure homologies and their relation. For this purpose, we define a bi-alignment to consist of two alignments (one based one sequence similarity, the other one based on structure similarity) that are related by a third alignment, which captures the shift events. All three constituent alignments contribute to a common score.

While bi-alignments have similarities to *combined sequence and structure alignments* (which also optimize a joint score for sequence and structure similarity), bi-alignments extend such models by supporting shift events explicitly. Combined sequence/structure alignments therefore can be interpreted as the limit case of bi-alignments where arbitrarily high shift penalties completely prohibit shift events. As important consequence, bi-alignments overcome the requirement of a consensus structure, which is the key assumption underlying combined sequence/structure alignments.

As their main purpose, bi-alignments provide a *coherent framework* to detect shift-like incongruences, i.e. a local “movement” of conserved structures relative to the underlying sequence. It is worth noting that the formal concept of bi-alignments is not tied to applications in structural biology. Instead, it can be seen as a way to quantify the effect of differences in scoring schemes that focus on different aspects of the same sequence. The only requirement for bi-alignments is a position-wise one-to-one correspondence between the two different representations of each input object.

In this contribution, we extend bi-alignments with linear costs to a more realistic model with *affine gap costs*. We will illustrate our algorithmic developments using protein sequences and their secondary structures as an example, because the position-wise annotation of a secondary structure elements fits well with the framework of sequence alignments. The (artificial) example in Fig. 1 shows that incongruence between sequence and secondary structure can indeed be caused a few well-place substitutions. It also shows that bi-alignments are capable, at least in principle, to reconcile incongruent sequence and structure homologies and to identify shift events.

A *bi-alignment* is formally defined as an alignment relating two, generally different, alignments of the same objects.

#### **Definition 1**

A *bi-alignment* $\mathbb {A}\cong (\mathbb {U},\mathbb {V},\mathbb {W})$ consists of two pairwise alignments $\mathbb {U}$ and $\mathbb {V}$ of the objects $\mathbf {a}$ and $\mathbf {b}$ and an alignment $\mathbb {W}$ of $\mathbb {U}$ and $\mathbb {V}$.

In Fig. 1, $\mathbb {U}$ is a sequence alignment (shown in the second row with the secondary structure annotation above and below the two sequences), while $\mathbb {V}$ is an alignment of the two respective secondary structures (shown in the second row with the two corresponding sequences between them). The columns of $\mathbb {U}$ and $\mathbb {V}$ are then aligned by $\mathbb {W}$. Since the pairwise alignment of two pairwise alignments is equivalent to a 4-way alignment, bi-alignments can be thought of as multiple alignments $\mathbb {A}\cong (\mathbb {U},\mathbb {V},\mathbb {W})$. The input objects $\mathbf {a}$ and $\mathbf {b}$ appear twice in $\mathbb {A}$, once regarded as sequence (represented by the one-letter amino acid codes) and once regarded as secondary structure (shown a position-wise glyphs). Bi-alignments therefore differ from “structure-aware” sequence alignments by replacing the *annotation* of sequence positions with a secondary structure features by an *alignment* of both the sequence and the string of structural features. Importantly, $\mathbb {A}\cong (\mathbb {U},\mathbb {V},\mathbb {W})$ completely determines the alignments of the sequences of $\mathbf {a}$ and $\mathbf {b}$ with their secondary structures (shown in the third row of Fig. 1 as the first and last pair of rows, respectively.) These alignments in general contain gaps that indicate how the conserved “consensus” structure is shifted compared to the sequence positions.

Assuming a *linear scoring model*, i.e. scores for $\mathbb {U}$, $\mathbb {V}$, and $\mathbb {W}$ that are additively composed from single column contributions, it can be shown that the 4-way alignment $\mathbb {A}$ is scored additively as well [12, 23]. Linear bi-alignment problems therefore can be exactly solved by dynamic programming [24, 25] in quartic time. In this contribution we are interested in bi-alignments that are scored with affine gap costs.

### Alignments as regular multi-tape grammars

To address this problem, it is helpful to describe the structure of alignments by multi-tape grammars, see e.g. [26] for a more detailed, formal discussion. In the simplest case, sequence alignments can be represented as regular grammars of the form $A\rightarrow Ac \mathrel {\big \vert }\epsilon$. The only non-terminal symbol *A* denotes a (pairwise) alignment, the terminal $\epsilon$ is the empty alignment, and the terminal *c* denotes an alignment column, which may be a (mis)match $${\left( {\begin{matrix}\bullet \\ \bullet \end{matrix}}\right) }$$, a deletion $${\left( {\begin{matrix}\bullet \\ -\end{matrix}}\right) }$$, or an insertion $${\left( {\begin{matrix}-\\ \bullet \end{matrix}}\right) }\}$$. Since alignments compare extant sequences rather than an ancestor/descendant pair, the two “indels” (insertion/deletion) are biologically indistinguishable and hence receive the same score. The grammar simply expresses the fact that alignment can be constructed step-by-step by adding a column to an alignment of prefixes. For linear scoring functions, the production $A\rightarrow Ac$ allows adding the score of *c* to the previously accumulated score of the alignment *A*. Denote by *M*(*x*) the *optimal* score of an alignment of the prefixes $\mathbf {a}[1..x_1]$ and $\mathbf {b}[1..x_2]$. As noted e.g. in [27, 28], the index vector of the penultimate column of the alignment is $x-c$, where $\bullet$ is interpreted as 1 and the gap character − as 0. The Needleman–Wunsch recursions [29] thus can be written in compact form (see also [24]) as

1 $$M(x) = \max _c M(x-c) + s(x,c) \quad \text {with}\quad M(0)=0\,.$$

Notably, in the non-affine case, the scoring function *s*(*x*, *c*) is completely determined by a single column.

*Affine gap cost.* While linear gap costs are not very realistic in sequence alignment [30], arbitrary gap costs algorithmically require an additional factor *O*(*n*) in running time [31, 32] and are difficult to parametrize in practice. The *affine gap cost* model serves as a useful and convenient compromise that is most often used in practice. Here, the opening and the extension of a gap are scored differently. It is therefore necessary to distinguish three different non-terminal $$A_{{\left( {\begin{matrix}\bullet \\ \bullet \end{matrix}}\right) }}$$, $$A_{{\left( {\begin{matrix}\bullet \\ -\end{matrix}}\right) }}$$, $$A_{{\left( {\begin{matrix}-\\ \bullet \end{matrix}}\right) }}$$ designating alignments that end in a (mis)match, deletion, and insertion column, respectively. Again one obtains a regular grammar with analogous productions of the form $A_c \rightarrow A_{c^{\prime}}c \mathrel {\big \vert }\epsilon$ for the three non-terminals. Denote by *M*(*x*; *c*) the optimal score of an alignment of the prefixes $\mathbf {a}[1..x_1]$ and $\mathbf {b}[1..x_2]$ with end column of type *c*. We can then write Gotoh’s well-known recursions [33] for pairwise affine gap cost alignment in the following compact form:

2 $$M(x;c) = \max _{c^{\prime}} M(x-c;c^{\prime}) + s(x,c^{\prime},c)$$

with initial conditions $$M(0,{\left( {\begin{matrix}\bullet \\ \bullet \end{matrix}}\right) })=0$$, $$M\left(0,{\left( {\begin{matrix}-\\ \bullet \end{matrix}}\right) }\right)=M\left(0,{\left( {\begin{matrix}\bullet \\ -\end{matrix}}\right) }\right)= -\infty$$. In principle this formulation accommodates any scoring function $s(x,c^{\prime},c)$ for which the column score depends on the gap pattern of the previous column. For instance, we could also score the closing of a gap separately.

Both the Needleman–Wunsch algorithm and the Gotoh algorithm run in $O(n^2)$ space and time. Recursion Eq. (1) also describes the dynamic programming algorithm for *k*-ary alignments [24, 25, 34, 35], which requires $O(n^k)$ space and time. The situation is more complicated, however, for affine gap costs. Sum-of-pairs scoring functions simply sum over the scores of all pairwise alignments contained in a given multiple alignment. Surprisingly, computing the optimal alignment of alignments with affine gap costs under the sum-of-pairs-model is NP-complete unless the number of sequences in the constituent alignments is bounded [36]. On the other hand, scoring models of the form of Eq. (2) are of practical interest in particular for $k=3$ [37–39].

In this contribution we show that the bi-alignment model with affine gap costs for the constituent alignments can be solved in polynomial time by dynamic programming. As we shall see, the recursions are of the form of Eq. (2) but require a subtle re-definition of *M*(*x*; *c*).

## Theory

### Bi-alignments

Recall that we define a bi-alignment as an alignment of alignments (Def. 1). It is well known that an alignment of alignments can be represented again as an alignment. This compositional structure of alignments is discussed formally in [40]. In our case, $\mathbb {A}$ is a 4-way alignment from which $\mathbb {U}$ (and $\mathbb {V}$) are obtained as “projections”, i.e. by extracting the corresponding pair of rows and removing all columns consisting of a pair of gap characters. The alignment $\mathbb {W}$, on the other hand, is obtained by considering each column in $\mathbb {U}$ and $\mathbb {V}$ as a single letter; and moreover interpreting the columns of the form $${\left( {\begin{matrix}-\\ -\end{matrix}}\right) }$$ (i.e. the ones that are removed in the projections to $\mathbb {U}$ and $\mathbb {V}$) as gap characters.

The Bi-Alignment Problem for two input sequences $\mathbf {a}$ and $\mathbf {b}$ consists in optimizing

3 $${\text{score}}(\mathbb {A}) = u(\mathbb {U}) + v(\mathbb {V}) + w(\mathbb {W})$$

with given scoring functions *u*, *v*, and *w*. The special case where *u*, *v*, and *w* are linear scoring functions has been discussed in [12, 23].

**Fig. 3:**
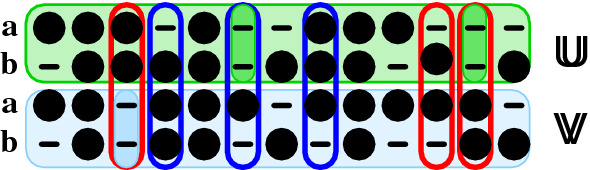
Shifts in a bi-alignment. The bi-alignment consists of two alignments $\mathbb {U}$ or $\mathbb {V}$ (colored horizontal boxes) of the pair of objects $\mathbf {a}$ and $\mathbf {b}$ that are aligned with each other two different ways i.e. w.r.t. to two different objective functions. Since the actual letters in $\mathbf {a}$ and $\mathbf {b}$ are irrelevant for definition of shifts, we distinguish only letters (filled circles) and gaps (dashes). Note that $\mathbf {a}$ and $\mathbf {b}$ may be represented by different alphabets in $\mathbf {U}$ and $\mathbf {V}$. Insertions and deletions in the alignment of alignments $\mathbb {W}$, i.e. the alignment of the columns of $\mathbb {U}$ with the columns of $\mathbb {W}$, are (highlighted by darker colors) correspond to all-gap columns in either $\mathbb {U}$ or $\mathbb {V}$. Aligned columns in $\mathbb {W}$ are shifts if the gap patterns in the upper pair and the lower pair differ. Colored outlines distinguish single (blue) and double shifts (red)

The alignment $\mathbb {W}$ of $\mathbb {U}$ and $\mathbb {V}$ describes the shifts distinguishing $\mathbb {U}$ and $\mathbb {V}$ in the following manner. First, consider a match column $\alpha$ of $\mathbb {W}$. It consists of a pair of columns with gap patterns $c(\alpha )$ and $d(\alpha )$, respectively. Using their numerical interpretation, we observe that

4 $$s(\alpha ):=|c_1(\alpha )-d_1(\alpha )|+|c_2(\alpha )-d_2(\alpha )|$$

measures whether none, one, or both input sequences are shifted relative to each other (Fig. 3). Insertions and deletions in $\mathbb {W}$ correspond to inserting an all-gap column $${\left( {\begin{matrix}-\\ -\end{matrix}}\right) }$$ into $\mathbb {U}$ or $\mathbb {V}$, respectively, and always lead to incongruences. We note, furthermore, that there is a one-to-one correspondence between the columns of $\mathbb {W}$ and the columns of the 4-way alignment $\mathbb {A}$. Thus we can count the number of shifts $s(\mathbb {A})=\sum _{\alpha \in \mathbb {A}} s(\alpha )$. The alignment $\mathbb {A}$ contains sub-alignments $\mathbb {A}^{(\mathbf {aa})}$ and $\mathbb {A}^{(\mathbf {bb})}$ of the first and second input sequence with itself. Let us denote the number of indels in these two projected alignments by $\delta _{\mathbf {a}}$ and $\delta _{\mathbf {b}}$, respectively.

#### **Lemma 2**

*If* $\mathbb {A}\cong (\mathbb {U},\mathbb {V},\mathbb {W})$ *is a bi-alignment of* $\mathbf {a}$ *and* $\mathbf {b},$ *then* $s(\mathbb {A})=\delta _{\mathbf {a}}+\delta _{\mathbf {b}}.$

#### Proof

For column $\alpha$ of $\mathbb {A}$ we write $\delta _{\mathbf {a}}(\alpha ):=|c_1(\alpha )-d_1(\alpha )|$ and $\delta _{\mathbf {b}}(\alpha ):=|c_2(\alpha )-d_2(\alpha )|$. Thus $\delta _{\mathbf {a}}(\alpha )=1$ if $\alpha$ is an indel column in the projected self-alignment of $\mathbf {a}$, and $\delta _{\mathbf {a}}(\alpha )=0$ if $\alpha$ is a (mis)match column. Note that all-gap columns are omitted in the projection and thus do not contribute to the indel count. Thus $\delta _{\mathbf {a}}=\sum _{\alpha \in \mathbb {A}} \delta _{\mathbf {a}}(\alpha )$ correctly counts the indels in $\mathbb {A}^{(\mathbf {aa})}$. An analogous equality holds for $\delta _{\mathbf {b}}$. A comparison with Eq. (4) completes the proof. $\square$

A natural scoring function for $\mathbb {W}$ is thus to penalize the total number of shifts, setting $w(\mathbb {A})=-\Delta s(\mathbb {A})$. This amounts to computing the shift contribution for each column $${\left( {\begin{matrix}c\\ d\end{matrix}}\right) }$$ of $\mathbb {A}$ as ${\text{shift}}(c,d)= -\Delta |c-d| = -\Delta \,(|c_1-d_1|+|c_2-d_2|)$.

### Bi-alignments with affine gaps costs

Lemma 2 provides an alternative interpretation in terms of a simple linear score for $\mathbb {A}^{(\mathbf {aa})}$ and $\mathbb {A}^{(\mathbf {bb})}$. We can therefore think of Eq. (3) as a restricted sum-of-pairs model in which only four of the six pairwise alignments in $\mathbb {A}$ contribute. In this picture it is natural to assume that the constituent alignments $\mathbb {U}$ and $\mathbb {V}$ are scored with affine gap costs. In the light of the NP-hardness result of [36] it is not at all obvious, however, that the bi-alignment problem with affine gap costs can be solved in polynomial time.

In order to address this problem, we first recall the language of multi-way alignments. The following statement is “folklore”, see e.g. [40]: every column of the 4-way alignment $\mathbb {A}$ is uniquely determined by

i. a four-dimensional index (*x*, *y*) identifying the prefixes $\mathbf {a}[1..x_1]$, $\mathbf {b}[1..x_2]$, $\mathbf {a}[1..y_1]$, and $\mathbf {b}[1..y_2]$ that are aligned up to the focal column.
ii. a gap pattern $(c,d)=((c_1,c_2),(d_1,d_2))$ specifying whether the entry in a column is a letter or a gap character.

The language of 4-way alignments is generated by the regular language $$A\rightarrow A{\left( {\begin{matrix}c\\ d\end{matrix}}\right) } \mathrel {\big \vert }\epsilon$$, where the non-terminal *A* denotes a bi-alignment and the terminals $${\left( {\begin{matrix}c\\ d\end{matrix}}\right) }$$ correspond to one of the 15 possible gap patterns in a column of elements (excluding the all-gap column). Note that $$c={\left( {\begin{matrix}-\\ -\end{matrix}}\right) }$$ and $$d={\left( {\begin{matrix}-\\ -\end{matrix}}\right) }$$ respectively correspond to an insertion and deletion in $\mathbb {W}$, while $$c,d\ne {\left( {\begin{matrix}-\\ -\end{matrix}}\right) }$$ corresponds to a match in $\mathbb {W}$. This regular language is sufficient for linear gap cost models [12, 23].

In order to handle affine gap costs for $\mathbb {U}$ and $\mathbb {V}$, we need to keep track of the gap patterns of the preceding alignment column in $\mathbb {U}$ and $\mathbb {V}$. This is *not* the same as considering the preceding column of $\mathbb {A}$ because gap patterns of the form $${\left( {\begin{matrix}{\left( {\begin{matrix}-\\ -\end{matrix}}\right) }\\ d\end{matrix}}\right) }$$ and $${\left( {\begin{matrix}c\\ {\left( {\begin{matrix}-\\ -\end{matrix}}\right) }\end{matrix}}\right) }$$ correspond to all-gap columns, which are removed in $\mathbb {U}$ or $\mathbb {V}$. Thus, we introduce a new notion of column type to address these ’preceding’ gap patterns of the sub-alignments.

#### **Definition 3**

The *end column type* (*p*, *q*) of a bi-alignment $\mathbb {A}\cong (\mathbb {U},\mathbb {V},\mathbb {W})$ consists of the gap pattern *p* of the last column of $\mathbb {U}$ and the gap pattern *q* of the last column of $\mathbb {V}$. The end column type of the empty alignment is left arbitrary.

**Fig. 4:**
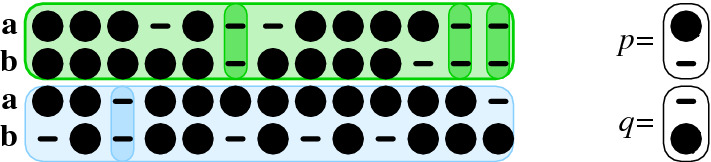
The *end column type* of an bi-alignment is defined by the last column of each of the constituent pairwise alignments of $\mathbf {a}$ and $\mathbf {b}$ that is not an all-gap column

The definition is illustrated in Fig. 4. Note that by construction, neither *p* nor *q* consist only of gaps.

Now, we define a column-wise scoring function that captures the alignment score with affine gap cost. It scores a single column of a bi-alignment $\mathbb {A}$, characterized by $${\left( {\begin{matrix}x\\ y\end{matrix}}\right) }$$ and $${\left( {\begin{matrix}c\\ d\end{matrix}}\right) }$$, depending on the end column type $${\left( {\begin{matrix}c^{\prime}\\ d^{\prime}\end{matrix}}\right) }$$ of the previous column. This function has the form

5 $$\begin{aligned} {\text{score}}\left({\left( {\begin{matrix}x\\ y\end{matrix}}\right) },{\left( {\begin{matrix}c^{\prime}\\ d^{\prime}\end{matrix}}\right) },{\left( {\begin{matrix}c\\ d\end{matrix}}\right) }\right)&= {\text{score}}_{\mathbb {U}}(x,c^{\prime},c)+{\text{score}}_{\mathbb {V}}(y,d^{\prime},d)+{\text{shift}}(c,d)\\ \text {with}{\text{score}}(x,c^{\prime},0)&={\text{score}}(y,d^{\prime},0)=0 \end{aligned}$$

Since ${\text{score}}(x,c^{\prime},0)$ and ${\text{score}}(y,d^{\prime},0)$, respectively, correspond to all-gap columns in $\mathbb {U}$ and $\mathbb {V}$, we observe that the sum of the $${\text{score}}\left({\left( {\begin{matrix}x\\ y\end{matrix}}\right) },{\left( {\begin{matrix}c^{\prime}\\ d^{\prime}\end{matrix}}\right) },{\left( {\begin{matrix}c\\ d\end{matrix}}\right) }\right)$$ over all columns of $\mathbb {A}$ equals

6 $$\begin{aligned} \sum _{(x,c)\in \mathbb {U}}&{\text{score}}_{\mathbb {U}}(x,c^{\prime},c) + \sum _{(y,d)\in \mathbb {V}} {\text{score}}_{\mathbb {V}}(y,d^{\prime},d) + \sum _{(c,d)\in \mathbb {W}} {\text{shift}}(c,d) \\&= u(\mathbb {U}) + v(\mathbb {V}) + {\text{shift}}(\mathbb {A}) \end{aligned}$$

Thus, Eq. (5) correctly scores the bi-alignment with general affine gap costs for both $\mathbb {U}$ and $\mathbb {V}$.

In order to derive a dynamic programming algorithm that solves the bi-alignment problem with this type of scoring function, we consider a decomposition of the search space in grammar form. The non-terminals $A_{(p,q)}$ correspond to bi-alignment with end column type (*p*, *q*). The terminals are the 15 possible column types of a 4-way alignment, which we write as $${\left( {\begin{matrix}p\\ q\end{matrix}}\right) }$$, with $$p,q\ne {\left( {\begin{matrix}-\\ -\end{matrix}}\right) }$$ as well as $${\left( {\begin{matrix}-\\ q\end{matrix}}\right) }$$ $${\left( {\begin{matrix}q\\ -\end{matrix}}\right) }$$ where the − in the latter is a shorthand for $${\left( {\begin{matrix}-\\ -\end{matrix}}\right) }$$. In addition, we write $\epsilon$ for the empty 4-way column.

#### **Lemma 4**

*The language of bi-alignments with fixed end column type is generated by the productions*

7 $$A_{(p,q)} \rightarrow A_{(p^{\prime},q^{\prime})}{\left( {\begin{matrix}p\\ q\end{matrix}}\right) } \mathrel {\big \vert }A_{(p,q^{\prime})}{\left( {\begin{matrix}-\\ q\end{matrix}}\right) } \mathrel {\big \vert }A_{(p^{\prime},q)}{\left( {\begin{matrix}p\\ -\end{matrix}}\right) } \mathrel {\big \vert }\epsilon$$

#### *Proof*

Consider an alignment $\mathbb {A}$ with last column (*c*, *d*) and end column type (*p*, *q*), and denote by $\mathbb {A}^{\prime}$ the alignment without the last column. If $$c,d\ne {\left( {\begin{matrix}-\\ -\end{matrix}}\right) }$$, i.e. the (mis)match case in $\mathbb {W}$, then $p=c$ and $q=d$ and $\mathbb {A}^{\prime}$ may have any end-column type. If $$c={\left( {\begin{matrix}-\\ -\end{matrix}}\right) }$$, corresponding to the insertion case in $\mathbb {W}$, $\mathbb {A}$ inherits the first component *c* of its end column type from the previous alignment $\mathbb {A}^{\prime}$. The other component is given by the second part of the last column, i.e. $d=q$. Thus the second component of the end column type of $\mathbb {A}^{\prime}$ is arbitrary. The case $$d={\left( {\begin{matrix}-\\ -\end{matrix}}\right) }$$, deletion in $\mathbb {W}$ analogously yields $d=q$ and an end column type $(p^{\prime},q)$ for the $\mathbb {A}^{\prime}$. $\square$

Note that this grammar would allow terminating with any end column type. This is undesirable since we would like the first column to be scored as it was preceded by a match column in both $\mathbb {U}$ and $\mathbb {V}$. This is easily implemented by an appropriate initialization for $x=y=0$, however.

#### **Definition 5**

Let $M_{p,q}(x,y)$ denote the optimal score of a 4-way alignment with end column type (*p*, *q*).

In order to enforce that empty alignment is treated as having end column type $$\left({\left( {\begin{matrix}\bullet \\ \bullet \end{matrix}}\right) },{\left( {\begin{matrix}\bullet \\ \bullet \end{matrix}}\right) }\right)$$, we set $$M_{\left({\left( {\begin{matrix}\bullet \\ \bullet \end{matrix}}\right) },{\left( {\begin{matrix}\bullet \\ \bullet \end{matrix}}\right) }\right)}(0,0)=0$$ and $M_{(c,d)}(0,0)= -\infty$ for $$(c,d)\ne \left({\left( {\begin{matrix}\bullet \\ \bullet \end{matrix}}\right) },{\left( {\begin{matrix}\bullet \\ \bullet \end{matrix}}\right) }\right)$$.

#### **Theorem 6**

*The matrices* $M_{p,q}$ *satisfy the recursion*

8 $$M_{(p,q)}(x,y) = \max {\left\{ \begin{array}{ll} \displaystyle \max _{\begin{array}{c} {p^{\prime}\ne 0}\\ {q^{\prime}\ne 0} \end{array}} M_{(p^{\prime},q^{\prime})}(x-p,y-q) + {\text{score}}\left({\left( {\begin{matrix}x\\ y\end{matrix}}\right) },{\left( {\begin{matrix}p^{\prime}\\ q^{\prime}\end{matrix}}\right) },{\left( {\begin{matrix}p\\ q\end{matrix}}\right) }\right) \\ \displaystyle \max _{p^{\prime}\ne 0} M_{(p^{\prime},q)}(x-p,y) + {\text{score}} \left({\left( {\begin{matrix}x\\ y\end{matrix}}\right) },{\left( {\begin{matrix}p^{\prime}\\ q\end{matrix}}\right) },{\left( {\begin{matrix}p\\ 0\end{matrix}}\right) }\right) \\ \displaystyle \max _{q^{\prime}\ne 0} M_{p,q^{\prime}}(x,y-q) + {\text{score}} \left({\left( {\begin{matrix}x\\ y\end{matrix}}\right) },{\left( {\begin{matrix}p\\ q^{\prime}\end{matrix}}\right) },{\left( {\begin{matrix}0\\ q\end{matrix}}\right) }\right) \end{array}\right. }$$

#### *Proof*

We first note that every column of $\mathbb {A}$ is either a (mis)match or an indel column w.r.t. $\mathbb {W}$. These correspond to the first three alternative productions in Eq. (7), and cover all alternatives. Since $${\text{score}}\left({\left( {\begin{matrix}x\\ y\end{matrix}}\right) },{\left( {\begin{matrix}c^{\prime}\\ d^{\prime}\end{matrix}}\right) },{\left( {\begin{matrix}c\\ d\end{matrix}}\right) }\right)$$ depends only on the current column and the end column type, we obtain the optimal score of an alignment $\mathbb {A}$ with end column type (*p*, *q*) and last column (*c*, *d*) as the optimal score of an alignment $\mathbb {A}^{\prime}$ with any of the matching column type plus the score $${\text{score}} \left({\left( {\begin{matrix}x\\ y\end{matrix}}\right) },{\left( {\begin{matrix}c^{\prime}\\ d^{\prime}\end{matrix}}\right) },{\left( {\begin{matrix}c\\ d\end{matrix}}\right) }\right)$$ for the last column. The grammar in Eq. (7) specifies which end column types match. Furthermore, we note that, in the match case, the indices $(x^{\prime},y^{\prime})$ of the last column of the alignment to the left are given by $x-p$ and $x-q$, where (*p*, *q*) is gap pattern on the last column of $\mathbb {A}$. Correspondingly we have $(x^{\prime},y^{\prime})=(x-p,y)$ for the insertion case and $(x^{\prime},y^{\prime})=(x,y^{\prime}-q)$ in the insertion case. Taken together, this established the correctness of the recursion. $\square$

As an immediate consequence we have

#### **Corollary 7**

*The bi-alignment problem with affine gap cost models for the two constituent alignments can be solved in* $O(n^4)$ *time and space.*

### Affine shift costs

While bi-alignment with affine gap cost and linear shift costs may be of the most obvious practical relevance, we also discuss two variations with affine shift costs. First of all, we clarify how to attribute affine shift cost in our bi-alignment scoring model.

Let’s take a step back to our original definition of the bi-alignment score (Eq. 3) and our previous suggestion to define the “shift” score component $w(\mathbb {A})$ as $-\Delta s(\mathbb {A})$, i.e. as a multiple of $s(\mathbb {A})$. Since the latter was defined as the number of gap columns in the alignments $\mathbb {A}^{(\mathbf {aa})}$ and $\mathbb {A}^{(\mathbf {bb})}$, this amounts to scoring shifts in a linear cost model, where every shift has a cost of $\Delta$ per column.

For affine shift costs, we take the view that every consecutive run of gap symbols in the pairwise alignments of the two copies of $\mathbf {a}$ and $\mathbf {b}$ represents one shift. This shift is scored in the same way as gaps are scored under affine gap cost, i.e. based on the shift opening cost $\Delta _o$ plus the shift extension cost $\Delta$ times the length of the shift (number of shift columns).

We first consider affine shift cost and non-affine (i.e. linear) gap cost. Since affine shifts are scored exactly in the same way as affine gaps, this situation is symmetric to the case of affine gap cost combined with linear shift cost. The corresponding bi-alignment problem can thus be solved efficiently by applying exactly the same idea as in our previous algorithm (Theorem 6), only now keeping track by *p* and *q* of the gap patterns in the respective alignments of rows $1 \& 3$ and $2 \& 4$. We immediately obtain

#### **Corollary 8**

*The bi-alignment problem with affine shift cost models (and linear gap cost) can be solved in* $O(n^4)$ *time and space.*

### Combining affine gap and shift costs

More remarkably, we can even solve the general case of affine gap cost and affine shift cost in polynomial time by dynamic programming. Essentially, we combine the ideas of the above two algorithms. Our algorithm follows a grammar with general decomposition

9 $$A_{p} \rightarrow A_{p^{\prime}}c$$

In order to evaluate affine gaps and affine shifts correctly at the same time, we need to know the last non-gap-only gap patterns of all four pairwise alignments of rows $1 \& 2$, $1 \& 3$, $2 \& 4$, and $3 \& 4$; thus, we utilize non-terminals $A_p$, for all *p* that encode the respective gap patterns $p=(p_{12}, p_{13}, p_{24}, p_{34})$. By the same argument as before, we can show this information to be sufficient to score shifts and gaps correctly in affine cost models for every possible last column *c*.

One keeps track of the correct gap patterns for all of the relevant pairwise alignments by setting the entries of $p^{\prime}$ as

10 $$p^{\prime}_{ij} := {\left\{ \begin{array}{ll} p_{ij} & c_{i}=-\,\text { and }\,c_{j}=-\\ {\left( {\begin{matrix}c_{i}\\ c_{j}\end{matrix}}\right) }&\text {otherwise} \end{array}\right. }$$

for $ij\in \{12,13,24,34\}$, depending on *p* and *c* in Eq. (9). For termination, we add the grammar rule:

11 $$A_{p^0} \rightarrow \epsilon$$

for $$p^0:= \left({\left( {\begin{matrix}\bullet \\ \bullet \end{matrix}}\right) }, {\left( {\begin{matrix}\bullet \\ \bullet \end{matrix}}\right) }, {\left( {\begin{matrix}\bullet \\ \bullet \end{matrix}}\right) }, {\left( {\begin{matrix}\bullet \\ \bullet \end{matrix}}\right) }\right)$$. This allows implicit accounting for gap and shift openings of respective gaps and shifts at the left end of alignment strings.

*Remark*s *about generalizations and complexity* Note that the existence of an efficient algorithm for general affine bi-alignment does not contradict the general hardness of multiple alignment with affine gap costs, even if it suggests the following generalization: Multiple (*k*-way) alignment with affine gap costs can be computed by dynamic programming following the above idea of keeping track of the right-most non-gap-only gap-patterns in all pairwise alignments. This requires considering $$\left( {\begin{array}{c}k\\ 2\end{array}}\right)$$ many pairwise gap patterns, each out of three possibilities $${\left( {\begin{matrix}\bullet \\ \bullet \end{matrix}}\right) },{\left( {\begin{matrix}\bullet \\ -\end{matrix}}\right) },{\left( {\begin{matrix}-\\ \bullet \end{matrix}}\right) }$$. The resulting DP-algorithm for *k*-way alignment thus needs exponentially many matrices in *k*.

In bi-alignments of two sequences, we need to consider only four gap patterns, two for the two alignments and two for the shifts between the sequence copies. That is, there are (at most) $3^4=81$ combinations, which have to be represented by different matrices for the DP algorithm. This gets a little more practical, since many of these combinations cannot occur in valid bi-alignments. For example, having gap patterns $${\left( {\begin{matrix}\bullet \\ \bullet \end{matrix}}\right) }$$ for both alignments of $\mathbf {a}$ and $\mathbf {b}$, rules out all patterns for the alignments of the copies that contradict having last columns $${\left( {\begin{matrix}\bullet \\ \bullet \\ \bullet \\ \bullet \end{matrix}}\right) }$$,$${\left( {\begin{matrix}\bullet \\ \bullet \\ - \\ -\end{matrix}}\right) }$$, or $${\left( {\begin{matrix}- \\ -\\ \bullet \\ \bullet \end{matrix}}\right) }$$. Consequently, we find only 51 consistent gap pattern combinations, while we can proof 30 combinations inconsistent due to an analogous argument as sketched above.

### Sub-additive gap costs

The affine gap cost model, despite its algorithmic convenience, has been criticized because empirical gap length distributions usually are power laws thus suggesting a logarithmic gap costs [41]. However, gap costs of the form $w(\ell )=a + b\ell + c\ln \ell$ seem to yield better alignments in practice [42]. Pairwise alignments with subadditive gap costs can be computed by dynamic programming, considering insertions and deletions of arbitrary length:

12 $$M(x_1,x_2)=\max {\left\{ \begin{array}{ll} M(x_1-1,x_2-1)+s(x_1,x_2) \\ \max _{\ell \ge 1} M(x_1-\ell ,x_2)+w(\mathbf {a}[x_1-\ell +1..x_1]) \\ \max _{\ell \ge 1} M(x_1,x_2-\ell )+w(\mathbf {b}[x_2-\ell +1..x_2]) \end{array}\right. }$$

This idea does not seem to generalize to bi-alignments. It is possible, however, to generalize the end column type. Instead of only distinguishing $${\left( {\begin{matrix}1\\ 1\end{matrix}}\right) }$$, $${\left( {\begin{matrix}1\\ 0\end{matrix}}\right) }$$, $${\left( {\begin{matrix}0\\ 1\end{matrix}}\right) }$$, we can make each of them length dependent. This allows us to write the end column types $\langle p,\ell \rangle$, where $\ell \ge 1$ is the length of the run of columns of type *p* at the end of the alignment. With this notation we can write

13 $$\begin{aligned}M_{\langle p,\ell \rangle }(x)&= M_{\langle p,\ell -1 \rangle }(x) + d(x,p,\ell ) \text { for } \ell \ge 1 \\ M_{\langle p,0\rangle }(x)&= \max _{p^{\prime}\ne p} M_{\langle p^{\prime},\ell \rangle }(x) \\ \end{aligned}$$

with initial condition $M_{\langle p,0\rangle }(0)=0$. Here $d(x,p,\ell )$ equals the match score *s*(*x*) for $$p={\left( {\begin{matrix}\bullet \\ \bullet \end{matrix}}\right) }$$. For deletions, $$p={\left( {\begin{matrix}\bullet \\ -\end{matrix}}\right) }$$, we have $d(x,p,\ell )= w(\mathbf {a}[x_1-\ell +1..x_1])- w(\mathbf {a}[x_1-\ell +1..x_1-1])$. The extensions of an insertion is scored by an analogous expression. The auxiliary entries $M_{\langle p,0\rangle }(x)$ are used to correctly score alignments in which the last column is different from the previous end gap pattern. This recursion runs in cubic time, but also requires cubic space (instead of quadratic space). For our purposes, however, it has the advantage that the score is again defined column-wise albeit at the expense of having to keep track of a linear instead of a constant number of end gap types. It generalizes to a recursion with four indices to compute the optimal bi-alignment.

## Computational results

We implemented the bi-alignment algorithm with affine gap cost (Corollary 7) in Python 3. For improved performance, we adapted time-critical parts of the code to the Python C-extension Cython with some carefully chosen static typing. The new implementation was based on our previous implementation for RNA bi-alignment with linear gap cost [12, 23]. Like the earlier version, it allows the user to limit the number of positions either sequence can be shifted to the left or right against its own structure by a constant $\lambda$. The restricted recursions, following in essence the idea of [34, 35], have time complexity of $O(n^2\lambda ^2)$ instead of the unrestricted, but often impractical complexity $O(n^4)$. In addition to efficient bi-alignment with affine gap cost, new features have been added to the software:

1. Protein sequences may be scored with an arbitrary, user-defined similarity matrix. The BLOSUM62 matrix [43, 44] is supplied as default.
2. Protein secondary structures are scored using a simple bonus (here, 800) for matched secondary structure.
3. The dynamic programming matrices are stored as sparse matrices to to limit space consumption to $O(n^2\lambda ^2)$ (compared to $O(n^4)$ space complexity of a hypothetical non-sparse implementation).
4. In case of ambiguity, simpler shifts are preferred (For example the bi-alignment of Fig 1 has co-optima with shift events in both sequences or shift events that shift longer sub-sequences).
5. Improved graphical output of bi-alignments. Figures 1 and 5 were produced using a Jupyter notebook that is included in the software distribution.
  **Fig. 5:**
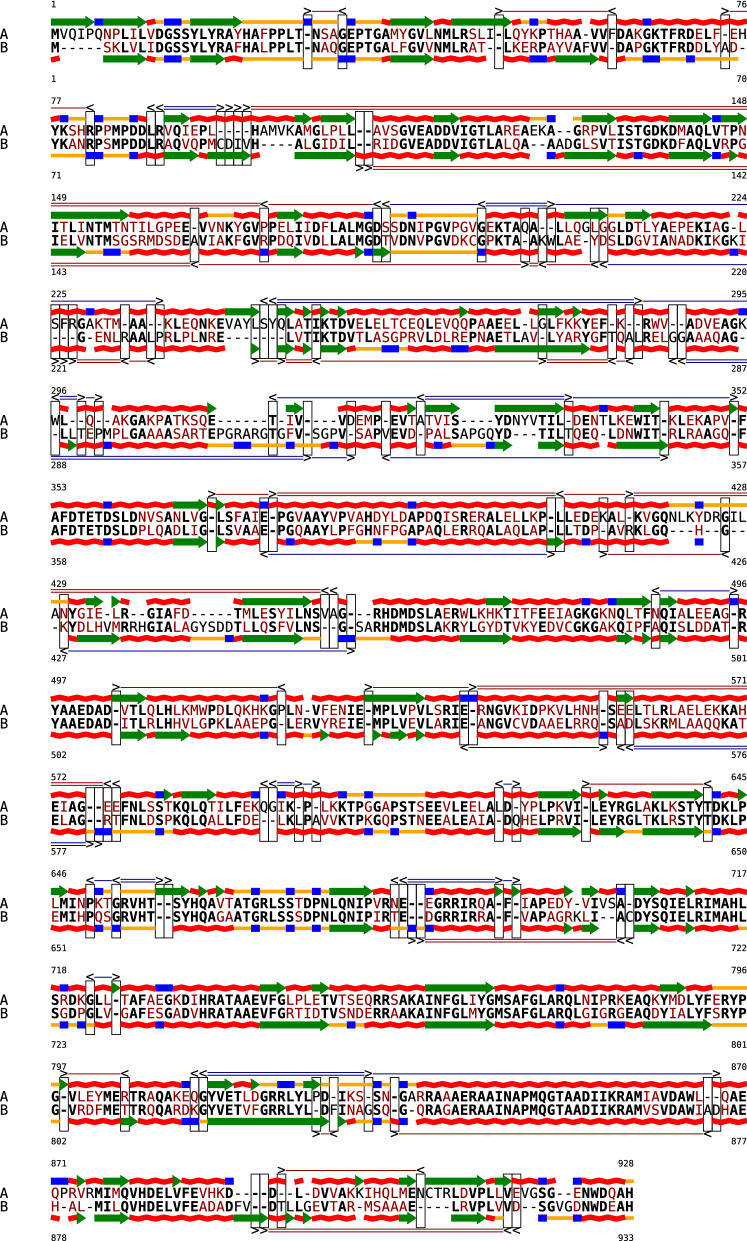
Bi-alignment of the proteins DNA Polymerase I of *Escherichia* (WP_016262675.1) and *Xanthomonas hortorum* (WP_095575020.1). We use the same representation as in Fig. 1
6. A flexible command line and Python interface is included.

As a proof of concept we generated optimal bi-alignments of DNA Polymerase I from *Escherichia* (length 928) and *Xanthomonas hortorum* (length 933), while allowing shifts of sequence against structure by up to two positions to the left or to the right in either protein ($\lambda =2$). On an Intel(R) Core(TM) i7-10810U CPU, this (single-threaded) computation took 37 min. Note that a simple banding strategy on insertions and deletions could dramatically speed up such computations, typically without sacrificing alignment quality. The analogous computation allowing only one shift positions ($\lambda =1$) was performed in 10.5 min. Due to filling 9 dynamic programming matrices and considering 15 recursion cases per entry, the same implementation still takes 26 s, if shifts are completely forbidden ($\lambda =0$).

Figure 5 shows the resulting bi-alignment for $\lambda =2$. For comparison, the results from $\lambda =1$ and $\lambda =0$ are given in Additional file 1. We chose a rather moderate shift cost $\Delta = -210$, compared to a bonus of 800 per structure match as well as gap extension and gap opening costs of $-50$ and $-200$, respectively. While we suspect that this parameter choice is too generous, it serves here to demonstrate that the algorithm readily predicts shifts that improve the compromise between primary and secondary structure alignment. The estimation of realistic shift costs is a non-trivial problem beyond the scope of this contribution.

## Concluding remarks

We have shown here that bi-alignments with affine gap cost models for both constituent alignments and linear shift costs can be computed in quartic time by dynamic programming. Moreover, limiting the number of shifts to a constant reduces the cost to quadratic space and time. This makes the detection of locally-confined shifts computationally feasible for sequences of with length of realistic proteins or mRNAs. While we have illustrated our algorithmic innovations here using amino acid sequences and protein secondary structures as an example, the algorithm and its implementation is applicable to any linear representation of monomer-wise features along a biopolymer. In can be used, for instance, directly as an extension of the linear-gap-cost bi-alignments of RNAs described in [12].

We have focused here on the analysis of optimization problem and development of the algorithm. In addition to cost models for the constituent alignments $\mathbb {U}$ and $\mathbb {V}$, a bi-alignment problem also requires the specification of the shift costs, i.e. the scoring model for $\mathbb {W}$. Even though the scoring systems for $\mathbb {U}$ and $\mathbb {V}$ are borrowed from other studies, the choice of appropriate shift parameters remains an open problem for future work. This is a difficult problem for two reasons: (i) There is, at present, no collection of test cases with known shifts of sequences versus secondary structure for either proteins or RNAs that could be used to optimize the parameters. (ii) A biologically sound survey of proteins should presumably use a more elaborate scoring model for secondary structure elements that distinguishes amino acid positions depending on the distance from the element’s ends. It stands to reason that the choice of the scoring model for the secondary structures would substantially influence estimates of the shift costs. Here, we are therefore content with a solution of algorithmic issues and a reference implementation. This provides the necessary tools for an in-depth empirical study of incongruent evolution of protein secondary structures in the future.

The formal framework of bi-alignments, Eq. (3), is much more general than the position-wise scoring models corresponding to regular multi-tape grammars. These were studied here because the corresponding optimization problems can be solved exactly by means of relatively simple dynamic programming algorithms. In a more general setting, one may want to consider $\mathbb {V}$ as an alignment of contact structures [45] or as an alignment of ordered sequences of 3D points, e.g. scored in terms of euclidean distances [46, 47]. This is of increasing practical interest as recent advances in protein folding [48, 49] provide access to high quality 3D structure predictions. The availability of accurately predicted protein structures of course also yields secondary structures, e.g. with the help of DSSP [50], which could be used for a systematic survey of incongruences in protein secondary structures. Alternatively, it seems promising to modify existing solutions to the protein structure alignment problems [51] to the corresponding bi-alignment problems. It is not obvious whether such a joint sequence and structure alignment problem implicitly contains a sequence-to-structure threading problem, which is known to be NP-complete [52]. In another forthcoming study, we are considering the corresponding problem for RNA secondary structures. In this case, the bi-alignment problem is amenable to a DP approach related to Sankoff’s algorithm for the simultaneous folding and alignment of RNAs [53].

In [12] we further generalized bi-alignments to poly-alignments comprising $k>2$ pairwise alignments $\mathbb {U}^{(i)}$, $1\le i\le k\ge 2$ that are connected by a *k*-way alignment $\mathbb {W}$. Each of the alignments $\mathbb {U}^{(i)}$ then describes one particular aspect of the sequence. In addition to the individual amino acids and secondary structure elements, these may represent comparisons of profiles of physico-chemical parameters. It is not difficult to see that the grammar Eq. (7) generalizes to this case by defining end gap types $(p_1,p_2,\dots p_k)$ with $$p_i\ne {\left( {\begin{matrix}-\\ -\end{matrix}}\right) }$$. The corresponding grammar then needs to consider all $2^k$ gap patterns for the last column of the *k*-way alignment $\mathbb {W}$. Optimal poly-alignments comprising *k* pairwise alignments with affine gap costs and additive cost contributions for the shifts between each pair of constituent alignments thus can be computed exactly in $O(n^{2k})$ space and time. Complementarily, one may consider alignments $\mathbb {U}$ and $\mathbb {V}$ of more than two sequences and their corresponding structures. The scoring of $\mathbb {W}$ then must accommodate more complex shift patterns, whose total number again increases exponentially in *k*. It is unlikely, therefore, that exact dynamic programming algorithms for these generalized problems will be practical. This begs the question whether poly-alignment problems can be approximated e.g. by progressive alignment schemes in a manner that is satisfactory from an applications point of view.

## Supplementary Information

**Additional file 1.** Bi-alignments with different choices of λ. Bi-alignments of the same data as in Fig. 5 using a more restrictive value (λ = 1) and a shift-free alignment (λ = 0). The latter corresponds to regular protein alignment with scores augmented by (mis)matches of the predicted secondary structure.

## Data Availability

Implementations of the algorithms used in this contribution are available as free software from https://github.com/s-will/BiAlign/releases/tag/v0.3. For easy installation, we provide packages bialign on the Conda channel bioconda and the Python Package Index PyPI, respectively.
