# Supplementary material for: Bi-alignments with affine gaps costs
Source: Algorithms Mol Biol. 2022 May 16;17:10. doi: 10.1186/s13015-022-00219-7 (PMC9109335; doi:10.1186/s13015-022-00219-7)

# Additional file 1

Alignment and bi-alignment with at most one shift of the proteins DNA Polymerase I of *Escherichia* (`WP_016262675.1`) and *Xanthomonas hortorum* (`WP_095575020.1`); for comparison to Fig. 4 of the main text.

## Alignment (Degenerated shift-free bi-alignment)

```
  1                                                                            78
A MVQIPQNPLILVDGSSYLYRAYHAFPPLTNSAGEPTGAMYGVLNMLRSLILQYKPTHA--AVVFDAKGKTFRDELFEHYK
B M-----SKLVLIDGSSYLYRAFHALPPLTNAQGEPTGALFGVVNMLRAT-LKERP--AYVAFVVDAPGKTFRDDLYADYK
  1                                                                            72

  79                                                                          158
A SHRPPMPDDLRVQIEPLHAMVKAMGLPLLAVSGVEADDVIGTLAREAEKAGRPVLISTGDKDMAQLVTPNITLINTMTNT
B ANRPSMPDDLRAQVQPMCDIVHALGIDILRIDGVEADDVIGTLALQAAADGLSVTISTGDKDFAQLVRPGIELVNTMSGS
  73                                                                          152

  159                                                                         232
A ILGPEE-VVNKYGVPPELIIDFLALMGDSSDNIPGVP--G--VGEK-TAQALLQGLGGLDTLYAEPEKIAGLSFRGAKTM
B RMDSDEAVIAKFGVRPDQIVDLLALMGDTVDNVPGVDKCGPKTAAKWLAEY-----DSLDGVIANADKIKGKIGENLRAA
  153                                                                         227

  233                                                                         304
A AAKLEQNKEVAYLSYQLATIKTDVELELTCEQLEVQQPAAEEL--L----GLFKKYEFKRWVADVEAGKWL--QAKGAKP
B LPRLPLNRELV-------TIKTDVTLASGPRVLDLREPNAETLAVLYARYG-FTQALRELGGAAAQAGLLTEPMPLGAAA
  228                                                                         299

  305                                                                         370
A ATK-------------S-QETIVVDEMPEVTATVISYDNYVTILDENTLKEWITKLEKAPVFAFDTETDSLDNVSANLVG
B ASARTEPGRARGTGFVSGPVSAPVEVDPALSAPG-QYDT-I-LTQE-QLDNWITRLRAAGQFAFDTETDSLDPLQADLIG
  300                                                                         375

  371                                                                         445
A LSFAIEPGVAAYVPVAHDYLDAPDQISRERALELLKPLLEDEKALKVGQNLKYDRGILANYGIELRGIAF-----DTMLE
B LSVAAEPGQAAYLPFGHNFPGAPAQLERRQALAQLAPLLTDPAVRKLGQHGKYD---L--HVMRRHGIALAGYSDDTLLQ
  376                                                                         450

  446                                                                         524
A SYILNSVAGRHDMDSLAERWLKHKTITFEEIAGKGKNQLTFNQIALEEAGRYAAEDADVTLQLHLKMWPDLQKHKGPLN-
B SFVLNSGSARHDMDSLAKRYLGYDTVKYEDVCGKGAKQIPFAQISLDDATRYAAEDADITLRLHHVLGPKLAA-EPGLER
  451                                                                         529

  525                                                                         602
A VFENIEMPLVPVLSRIERNGVKIDPKVLHNHSEELTLRLAELEKKAHEIAGEEFNLSSTKQLQTILFEKQGIK-P-LKKT
B VYREIEMPLVEVLARIEANGVCVDAAELRRQSADLSKRMLAAQQKATELAGRTFNLDSPKQLQALLFD--ELKLPAVVKT
  530                                                                         607

  603                                                                         682
A PGGAPSTSEEVLEELALDYPLPKVILEYRGLAKLKSTYTDKLPLMINPKTGRVHTSYHQAVTATGRLSSTDPNLQNIPVR
B PKGQPSTNEEALEAIADQHELPRVILEYRGLTKLRSTYTDKLPEMIHPQSGRVHTSYHQAGAATGRLSSSDPNLQNIPIR
  608                                                                         687

  683                                                                         762
A NEEGRRIRQAFIAPEDYVIVSADYSQIELRIMAHLSRDKGLLTAFAEGKDIHRATAAEVFGLPLETVTSEQRRSAKAINF
B TEDGRRIRRAFVAPAGRKLIACDYSQIELRIMAHLSGDPGLVGAFESGADVHRATAAEVFGRTIDTVSNDERRAAKAINF
  688                                                                         767

  763                                                                         841
A GLIYGMSAFGLARQLNIPRKEAQKYMDLYFERYPGVLEYMERTRAQAKEQGYVETLDGRRLYLPD-IKSSNGARRAAAER
B GLMYGMSAFGLARQLGIGRGEAQDYIALYFSRYPGVRDFMETTRQQARDKGYVETVFGRRLYL-DFINAGSQGQRAGAER
  768                                                                         846

  842                                                                         914
A AAINAPMQGTAADIIKRAMIAVDAWL--QAEQPRVRMIMQVHDELVFEVHKD----DL-DVVAKKIHQLMENCTRLDVPL
B AAINAPMQGTAADIIKRAMVSVDAWIADHAEH-AL-MILQVHDELVFEADADFVDTLLGEVTARM-SAAAE----LRVP-
  847                                                                         918

  915          928
A LVEVGSG--ENWDQAH
B LV-VDSGVGDNWDEAH
  919          933
```

# Bi-alignment with at most one shift of either sequence in either direction

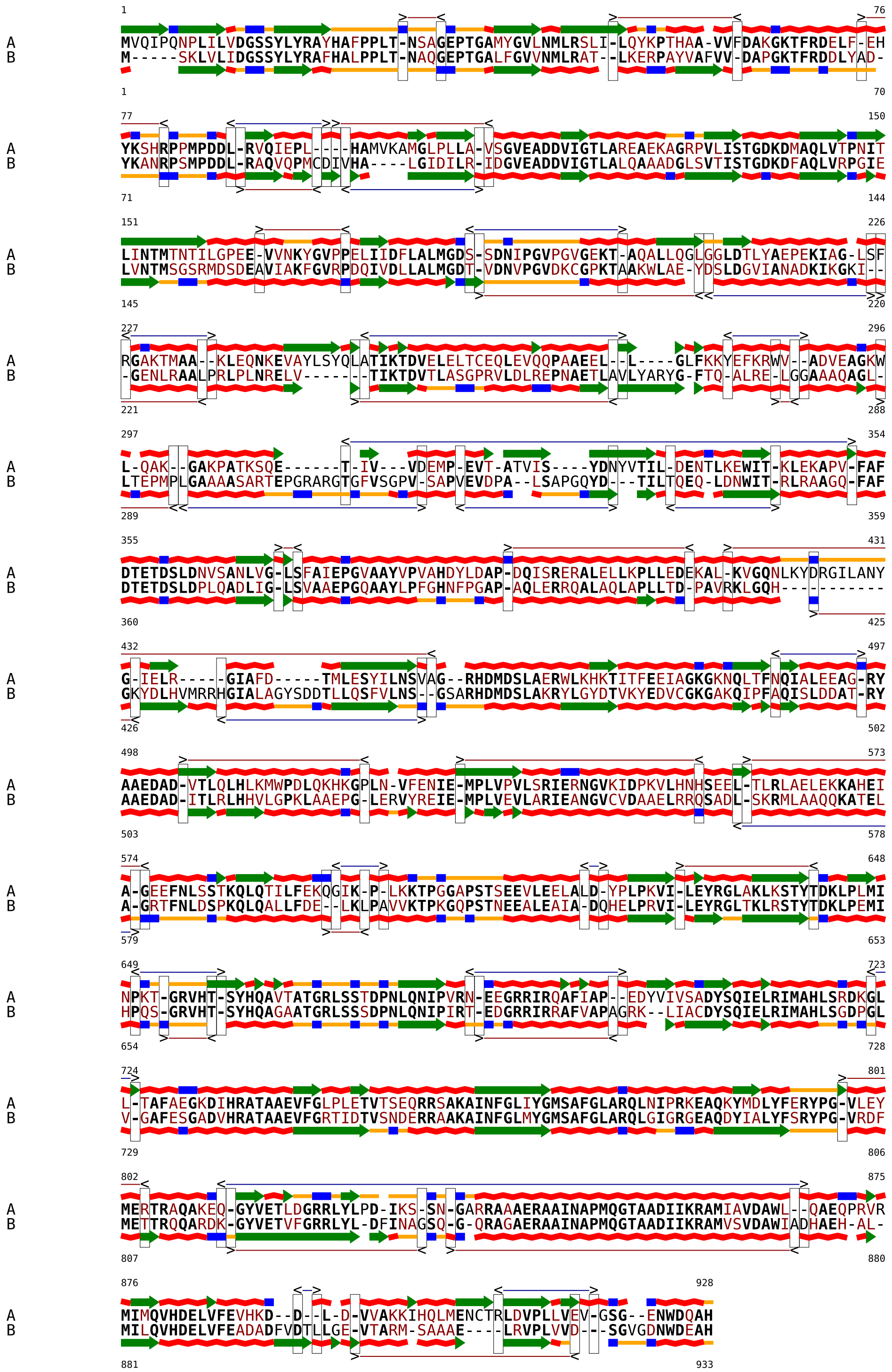

Supplement: Supplementary file 1 — Additional file 1. Bi-alignments with different choices of λ. Bi-alignments of the same data as in Fig. 5 using a more restrictive value (λ = 1) and a shift-free alignment (λ = 0). The latter corresponds to regular protein alignment with scores augmented by (mis)matches of the predicted secondary structure. [file 13015_2022_219_MOESM1_ESM.pdf]
